# Creation of Lung-Targeted Dexamethasone Immunoliposome and Its Therapeutic Effect on Bleomycin-Induced Lung Injury in Rats

**DOI:** 10.1371/journal.pone.0058275

**Published:** 2013-03-14

**Authors:** Xue-Yuan Chen, Shan-Mei Wang, Nan Li, Yang Hu, Yuan Zhang, Jin-Fu Xu, Xia Li, Jie Ren, Bo Su, Wei-Zhong Yuan, Xin-Rong Teng, Rong-Xuan Zhang, Dian-hua Jiang, Xavier Mulet, Hui-Ping Li

**Affiliations:** 1 Department of Respiratory Medicine, Shanghai Pulmonary Hospital, Tongji University School of Medicine, Shanghai, China; 2 Institute of Nano and Bio-polymeric materials, Tongji University, Shanghai, China; 3 Centrol Laboratory of Shanghai Pulmonary Hospital, Tongji University School of Medicine, Shanghai, China; 4 Department of Pathology, Shanghai Pulmonary Hospital, Tongji University School of Medicine, Shanghai, China; 5 Division of Pulmonary, Allergy, and Critical Care Medicine, Department of Medicine, Duke University School of Medicine, Durham, North Carolina, United States of America; 6 CSIRO Materials Science and Engineering, Clayton, Victoria, Australia; Clermont Université, France

## Abstract

**Objective:**

Acute lung injury (ALI), is a major cause of morbidity and mortality, which is routinely treated with the administration of systemic glucocorticoids. The current study investigated the distribution and therapeutic effect of a dexamethasone(DXM)-loaded immunoliposome (NLP) functionalized with pulmonary surfactant protein A (SP-A) antibody (SPA-DXM-NLP) in an animal model.

**Methods:**

DXM-NLP was prepared using film dispersion combined with extrusion techniques. SP-A antibody was used as the lung targeting agent. Tissue distribution of SPA-DXM-NLP was investigated in liver, spleen, kidney and lung tissue. The efficacy of SPA-DXM-NLP against lung injury was assessed in a rat model of bleomycin-induced acute lung injury.

**Results:**

The SPA-DXM-NLP complex was successfully synthesized and the particles were stable at 4°C. Pulmonary dexamethasone levels were 40 times higher with SPA-DXM-NLP than conventional dexamethasone injection. Administration of SPA-DXM-NLP significantly attenuated lung injury and inflammation, decreased incidence of infection, and increased survival in animal models.

**Conclusions:**

The administration of SPA-DXM-NLP to animal models resulted in increased levels of DXM in the lungs, indicating active targeting. The efficacy against ALI of the immunoliposomes was shown to be superior to conventional dexamethasone administration. These results demonstrate the potential of actively targeted glucocorticoid therapy in the treatment of lung disease in clinical practice.

## Introduction

Glucocorticoids are steroidal hormones with strong anti-inflammatory and immunosuppressive actions, which are widely used in clinical practice. Long-term systemic steroid therapy is routinely administered for many respiratory diseases, including acute lung injury/acute respiratory distress syndrome (ALI/ARDS) and interstitial pneumonia, bronchial asthma, sarcoidosis, and etc. [1], [2], [3]. Acute lung injury/Acute respiratory distress syndrome (ALI/ARDS) [4] are severe form of hypoxic lung disease due to many complicated causes and lead to a large number of deaths worldwide. They are defined clinically by gas exchange and chest radiographic abnormalities which occur shortly after a known predisposing injury and in the absence of heart failure. Acute respiratory distress syndrome (ARDS) represents the more severe end of the spectrum of this condition in which there are widespread inflammatory changes throughout the lung, usually accompanied by aggressive fibrosis in later stage. The common pathological feature of ALI/ARDS is diffused alveolar inflammation which lead to severe hypoxia and mortality in more than 70% of cases [5]. Animal models of acute lung injury (ALI) have contributed significantly to our understanding of the pathogenesis and pathophysiology of the clinical syndrome of ALI/ARDS [6]. Bleomycin (BLM) is a chemotherapeutic drug used for a variety of human malignancies treatment. But its benefits are limited by severe side effect of inducing pneumonitis and progressing to fibrosis [7]. Therefore, bleomycin is usually used in establishing acute lung injury and pulmonary fibrosis models in vivo [8].This animal model has diffused alveolar inflammation after with bleomycin from day 3 to 14, and then gradually progress to fibrosis. The model shows the features of early inflammation and later fibrosis. The model standardizes and reproduces well. Hence, it is a good animal model of acute lung injury, we used it to explore the effect of our new lung targeting agent.

Glucocorticoids have been used for treatment of ALI/ARDS for many years. However, systemic long-term or high-dose administration of glucocorticoids is often accompanied by adverse effects, disability and even life-threatening outcomes [3], [4], [9]. There is therefore an important unmet clinical need to reduce the severe side-effects of these glucocorticoids.

Harnessing advanced drug delivery techniques such as targeted delivery of therapeutic for such steroidal treatments holds great potential. Active targeting of drug delivery vehicles to a specific lesion can be achieved through coupling an antibody or antibody fragment to liposomes (known as immunoliposomes) [10], [11]. Liposomes have attracted considerable attention as drug delivery carriers because of their biocompatible and non-toxic nature which protects their cargo from degradation by plasma enzymes, and can enhance transports of their load through biological membranes [12], [13].Advantages of immunoliposome drug delivery vehicles also include reduced toxicity and adverse effects, as well as pharmacokinetic improvements such as a potential increase in half-life [14], [15].

Surfactant protein A(SP-A) was the first pulmonary surfactant protein to be identified. It is synthesized and released by type II alveolar epithelial cells. SP-A is rarely expressed outside lung tissue, but is highly expressed in the lung, indicating high lung-specificity. SP-A has been used as a classical indicator for identifying the origins of cells used in pathology [16], [17], [18]. We, therefore, selected SP-A polyclonal antibody as the lung-specific targeting agent to prepare dexamethasone(DXM)loaded immunoliposome (NLP) (SPA-DXM-NLP).The present study used pulmonary surfactant protein A (SP-A) antibody as a targeting agent to prepare a lung-specific dexamethasone sodium phosphate (DXM) immunoliposome. Proof-of-concept, was established by investigating the therapeutic effect of these immunoliposomes on acute lung injury in a rat model of lung disease.

## Materials and Methods

### Animals

All animal experiments were performed using Male Sprague Dawley SPF rats, 4 to 5 weeks old, weighing 90±10 g. The rats were, purchased from the SLAC Laboratory Animal Ltd., Co. (Shanghai, China). All animal experiments were approved by the Institutional Animal Ethics Committee for Experimentation on Animals of Tongji University.

### Preparation and Characteristics of DXM-NLP

#### Preparation of DXM-NLP

DXM-NLP was prepared through thin lipid film hydration combined with extrusion [14]–[19]. Soy lecithin, cholesterol and 1,2-distearoyl-sn-glycero-3- phosphoethanolamine-N-[maleimide (polyethylene glycol)-2000] (DSPE-PEG2000) (Avanti Polar Lipids Inc., USA) were dissolved at a molar ratio of 1.8∶1:0.2 in chloroform in a round-bottom flask. A thin lipid film was obtained by removing the solvent by rotating the flask at 120 r/min in a 50°C water bath. This yielded an evenly distributed transparent thin film layer on the flask wall. The lipid membranes were then hydrated by the addition of 1 ml of dexamethasone sodium phosphate (1 mg/ml) (Sigma, USA) dissolved in Phosphate Buffered Saline. This was incubated in a round-bottom flask in a water bath at 50°C and spun at 120 r/min for 2 h. The prepared liposome suspensions of were stored at room temperature for 2 h. The suspensions were then gradually passed through the 400-nm, 200-nm and 100-nm polycarbonate membrane filters to create liposomes with uniform size distribution. Thirteen extrusions using a Liposofast™ (Avestin; Ottawa, Canada) were performed for each membrane in order to formulate the DXM-NLP suspension.

#### Morphological observation and determination of particle size

The DXM-NLP suspension was diluted 50 times with physiological saline, stained with 1% uranyl acetate and observed under a JEM-1230 transmission electron microscope (JEOL Inc., USA). The suspension samples were diluted with distilled water and particle sized was determined by dynamic light scattering using a Zetasizer 3000 Hs Particle Size analyzer (Malvern Instruments Ltd., Co., UK).

#### Determination of encapsulation efficiency and stability

The encapsulation efficiency of DXM-NLP was determined using an internal standard method [20] in a HP1100 High Performance Liquid Chromatograph system (HPLC) (Agilent Technologies, USA). Chromatography was undertaken using a Platisil ODS (250 mm×4.6 mm, 5 µm; Dikma, USA) column with the following parameters. The flow phase used 0.34% potassium dihydrogen phosphate:methanol (35∶65); the column temperature was 30°C; flow velocity was 1 mL/min; detection wavelength was 240 nm; sample volume was 20 µL; and hydrocortisone acetate (National Institutes for Food and Drug Control, China) was used as an internal standard.

**Figure 1:**
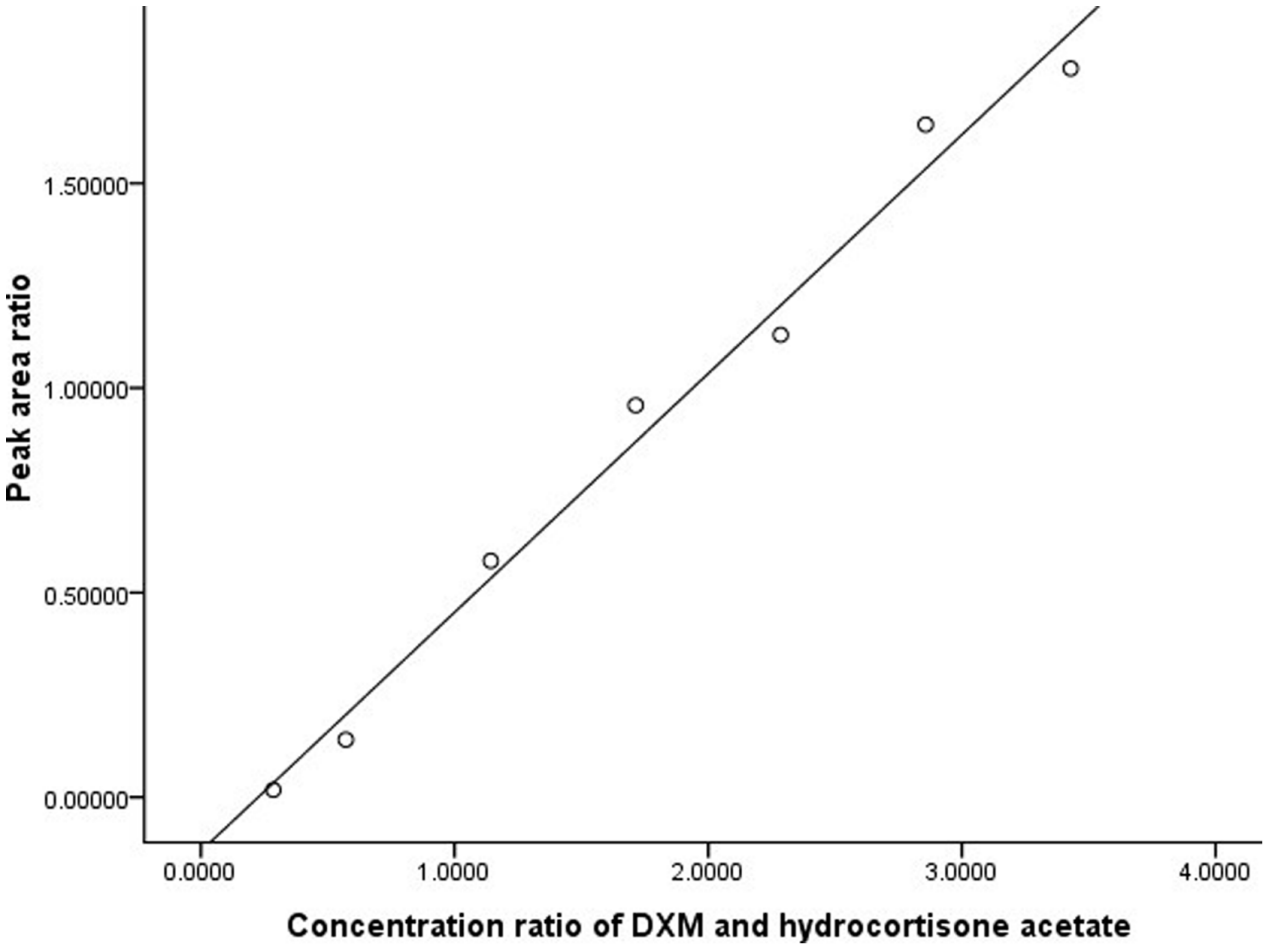
Linear regression of concentration ratio vs peak area ratio between DXM and hydrocortisone acetate. The standard curve equation was: Y  = 0.59065X-0.1441, r  = 0.9991.The ratio (X) of dexamethasone sodium phosphate to hydrocortisone acetate showed a good linear relationship to the peak area ratio (Y).

The ratio (*X*) of dexamethasone sodium phosphate (8 to 96 µg/mL) to hydrocortisone acetate (28 µg/mL) showed a good linear relationship to the peak area ratio (*Y*). The standard curve equation was: *Y*  = 0.59065*X*-0.1441, *r*  = 0.9991. (Figure 1).

Liposome suspensions (0.5 mL) were sampled, placed in an ultrafiltration tube (10 kD), and centrifuged at 10000 r/min for 30 min to separate the dexamethasone loaded in the liposomes from the unbound dexamethasone. The concentration of free dexamethasone sodium phosphate in ultrafiltrates was determined using HPLC. Liposome suspensions (0.5 mL) were dissolved in methanol to break up the liposomes and release the liposome-loaded dexamethasone. The resulting free dexamethasone sodium phosphate from the liposome fraction could then be quantified.

To assess the stability of these particles in long term storage, some of the DXM-NLP suspensions were divided into three and stored in 4°C. The stability of the particles in storage was determined by estimating the dexamethasome encapsulation efficiency after 1, 2, and 4 weeks.

#### Preparation of SP-A polyclonal antibody-coupled DXM-NLP (SPA-DXM-NLP)

N-succinimidyl-3-(2-pyridyldithiol) propionate (SPDP; Calbiochem, Germany) was used as a crosslinking agent to couple the antibody with the liposome and prepare the immunoliposome [21]. DXM-NLP was prepared using soy lecithin, cholesterol, DSPE-PEG2000 and DSPE-PEG2000-PDP [Avanti Polar Lipids Inc, USA] at a molar ratio of 1.8∶1:0.15∶0.05. SPDP ethanol solution (20 µmol/mL SPDP and SP-A antibody at a molar ratio of 20∶1, with an ethanol concentration <5%) was added into SP-A antibody solution (Santa Cruz, USA) (antibody : lecithin ratio: 2 µg : 1 µmol). The solutions were mixed evenly, and placed at room temperature for 30 min. The mixture was transferred to an ultrafiltration tube (10 kD) and centrifuged at 3500 r/min for 10 min. This was followed by the addition of 0.2 mol/L acetic acid-sodium acetate buffer solution (pH 5.5). Dithiothreitol solution (2.5 mol/L formulated by 0.2 mol/L acetic acid-sodium acetate buffer solution) was added to the mixture, mixed evenly, and placed at room temperature for 30 min. Citric acid-phosphate buffer solution (pH 7.0) was added, and then the solutions were centrifuged three times at 3500 r/min for 5 min to remove free dithiothreitol. The antibody solution in the ultrafiltration tube was collected and mixed with the DXM-NLP suspensions (containing DSPE-PEG2000-PDP), and stirred at room temperature overnight to obtain SPA-DXM-NLP.

The reaction of antibodies with SPDP results in the production of thiol. The resulting disulfide bonds in antibody derivatives can be reduced using dithiothreitol. In this process the antibody and liposome-containing PDP group are mixed, and the aromatic pyridinium thiol which has poor stability, is replaced by the aliphatic thiol at the site of the disulfide bond. This results in the coupling of antibody with liposomes via the disulfide bond [21].

### Tissue Distribution of SPA-DXM-NLP in Rats

#### Animals and groups

Male Sprague Dawley rats were randomly assigned to SPA-DXM-NLP, DXM-NLP and DXM groups. SPA-DXM-NLP, DXM-NLP and dexamethasone sodium phosphate were administered via tail vein injection at 2 mg/kg. Five rats in each group were anesthetized using ether at each of the following time points (0.25, 0.5, 1, 2, 4, 8 and 12 h) post-injection. Blood was collected via the orbital sinus. Ethylenediaminetetraacetic acid (EDTA) was used as an anti-coagulant, and the plasma was isolated by centrifugation at 10000 rpm for 5 minutes. Heart, liver, spleen, lung and kidney tissues were washed with saline solution, dried with filter paper and weighed. The plasma and tissues were stored at −20°C for subsequent experiments. The concentration of dexamethasone sodium phosphate in plasma and tissues was determined using HPLC with an external standard method.

#### Determination of Dexamethasone tissue concentration

Dexamethasone concentration was established using a similar protocol to that described earlier in this manuscript, using a Platisil ODS (250 mm×4.6 mm, 5 µm) chromatographic column. The flow phase included 0.34% potassium dihydrogen phosphate:methanol (42∶58). The column temperature was 30°C; flow velocity was 1 mL/min; detection wavelength was 240 nm and sample volume was 20 µL.

#### Pre-treatment of plasma and tissue samples

The heart, liver, spleen, lung and kidney tissues of rats were weighed, and formulated into 200 mg/mL tissue homogenates using saline solution. Plasma or tissue homogenates (0.5 mL) were collected, added to 1 mL methanol, spun for 3 min, and centrifuged at 12000 r/min for 10 min. The supernatant was collected and dried at 65°C in a Thermostat Metal Bath (CHB-100, Japan). The residues were dissolved in 0.2 mL methanol. They were then spun for 3 min and centrifuged at 12000 r/min for 10 min. Supernatant (20 µL) was sampled and dexamethasone levels assessed.

#### Assessment of tissue targeting

Peak concentration ratio (*C*_*e*_) and relative rate of uptake (*R*_*e*_) were used to investigate the targeting of SPA-DXM-NLP and DXM-NLP in different rat tissues. The following equations were used:
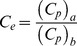
where *C*_*p*_ was drug peak concentration;‘_*a*_’ represented drug distribution in a tissue following injection with SPA-DXM-NLP or DXM-NLP;‘_*b*_’ indicated drug distribution in the same tissue following injection with dexamethasone sodium phosphate.



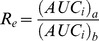
whereAUC_i_ was the area under concentration-time curve of tissue ‘*_i_’* calculated from the concentration-time curve using trapezoidal area method; ‘_*a*_’and ‘_*b*_’ are as described above.

### Therapeutic Effect of SPA-DXM-NLP on Bleomycin-induced Lung Injury in Rats

#### Animals and groups

Male Sprague Dawley rats were randomly assigned into six groups of 10 rats. Rats in groups A to E were endotracheally injected with 5 mg/kg bleomycin (Nippon Kayaku Co., Ltd., Japan) to establish lung injury. Rats in control Group F were endotracheally injected with the same volume of saline solution. Following the creation of animal models, Group A received SPA-DXM-NLP (1 mg/kg dexamethasone sodium phosphate) intravenously. once daily; Group B received SPA-DXM-NLP (0.5 mg/kg dexamethasone sodium phosphate) i.v. once daily; Group C received DXM-NLP (1 mg/kg dexamethasone sodium phosphate) i.v. once daily; and Group D received DXM (1 mg/kg dexamethasone sodium phosphate) i.v. once daily. The same volume of saline was given intravenously to rats in Group E and control group F.

Each group was divided into 1-week and 2-week intervention subgroups, with five rats in each subgroup. Rats were sacrificed after 1 or 2 weeks according to their subgroup allocation.

Another six groups of Male Sprague Dawley rats were grouped and administrated in the same method, but raised in the environment exposed to microorganism. We use these rats to do bacterial and fungal culture in broncho alveolar lavage fluid (BALF) separately.

#### Pathological observation of lung tissues

The middle lobe of right lung was fixed by infusing 10% formaldehyde solution in the same pressure, and the inflation of lung were kept uniform and then, embedded in paraffin wax, cut into sections and stained with hematoxylin-eosin (H&E). Pathological tissue changes in tissues were observed using optical microscopy. Lung injury, based on infiltration of inflammatory cells, pulmonary interstitial and alveolar edema, damage to alveolar structure and degree of fibrosis was assessed using the grading system reported by Szapiel *et al* [22]. And ALI was scored as follows [23]: a) alveolar congestion, b) hemorrhage, c) infiltration or aggregation of neutrophils in airspace or vessel wall, and d) thickness of alveolar wall/hyaline membrane formation. Each item was scored on a 5-point scale as follows: 0 minimal damage,1 mild damage, 2 moderate damage, 3 severe damage, and 4 maximal damage. Repeated-measures data were statistically analyzed using repeated-measures analysis of variance(ANOVA).

#### Bacterial and fungal culture in broncho alveolar lavage fluid (BALF)

Bronchial alveolar lavage was performed four times in the left lung using 1 mL saline, The broncho alveolar lavage fluid (BALF) was collected and the recovery volume recorded. The BALF was centrifuged at 3000 r/min for 15 min, and the supernatant was stored at −70°C prior to bacterial and fungal culture.

#### Determination of TNF-α and TGF-β1 levels in BALF

The protein levels of TNF-α and TGF-β1 in BALF were determined using enzyme-linked immunosorbent assay (ELISA, R&D Systems,US).

### Observation on Animal Survival

Male Sprague Dawley were randomly assigned to one of six groups of 12 rats. Rats in Groups A to and E were endotracheally injected with 10 mg/kg bleomycin to establish lung injury and rats in Group F received saline. The survival of rats in each group was compared within 8 weeks of intervention.

### Statistical Analysis

All statistical analyses were performed using Statistical Package for the Social Sciences (SPSS) version 16.0 (SPSS Inc., Chicago, IL, USA). Continuous data were described as means and standard deviations (± SD). Differences within groups were analyzed using one-way analysis of variance (ANOVA) and t-tests were used to analyze between group differences. Linear correlations were analyzed using F-tests. Differences in proportions and comparisons of rank data were tested using the chi-square test. The log-rank test was used to compare the survival curves. *P*-values >0.05 were considered statistically significant.

## Results

### Preparation and Characterisation of DXM-NLP

**Figure 2:**
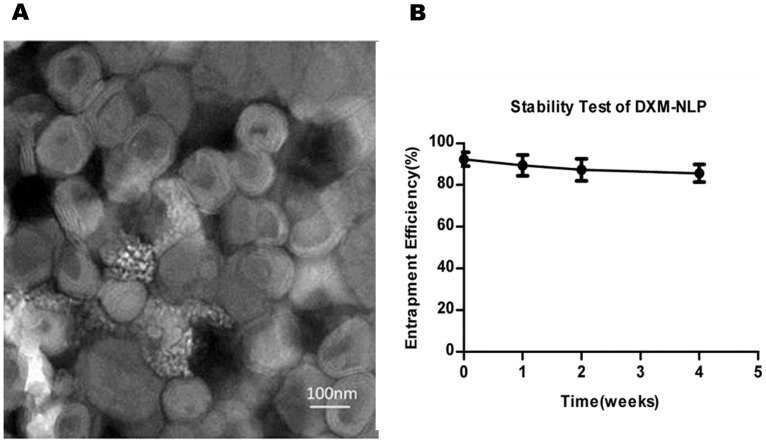
Characters of dexamethasone sodium phosphate nano-liposome(DXM-NLP). A). Micrograph showing dexamethasone sodium phosphate nano-liposome visualized by transmission electron microscope (×80000). Liposome particles were spherical and of approximately equal size. B) Stability of dexamethasone sodium phosphate nano-liposomes(DXM-NLP). DXM-NLP was prepared and stored at 4°C. No significant reduction of encapsulation efficiency was found after 4 weeks.

Transmission electron microscopy showed that DXM-NLP particles were spherical, and generally of equal size (Figure 2A). The mean particle size, measured using dynamic light scattering was 136±38 nm.

The efficiency of dexamethasone sodium phosphate encapsulation with DXM-NLP was (92±3%). There was no significant reduction in encapsulation efficiency in liposome suspensions that had been stored at 4°C for 4 weeks, suggesting that the particles were stable (Figure 2B).

### Distribution of SPA-DXM-NLP in Rat Tissue

#### Standard curve of dexamethasone sodium phosphate in plasma and tissue homogenates

Different concentrations of dexamethasone sodium phosphate solutions were added and mixed with plasma, heart, liver, spleen, lung and kidney homogenates, to formulate different standard solutions. Linear regression analysis was performed to define the relationship between chromatographic peak area and dexamethasone sodium phosphate concentrations. Chromatographic analysis showed that dexamethasone sodium phosphate was well separated from impurities in plasma and tissue homogenates. The regression equations for the standard curves in the plasma and tissue homogenates are shown in Table 1.

**Table 1:** Linear regression analysis of DXM concentrations (X) and peak areas (Y) in different samples.

Sample	Regressive Equation	Correlation Linear range coefficient (*r* ^2^)	Linear range (µg.mL^−1^)
Plasma	Y = 26996.875+98954.435X	0.997	0.0625-4
Lung	Y = 9580.224+55144.225X	0.993	0.0625-4
Liver	Y = 10744.63+46594.667X	0.997	0.0625-4
Spleen	Y = 1508.745+78723.915X	0.999	0.0625-4
Kidney	Y = 7356.32+77022.41X	0.996	0.0625-4
Heart	Y = 9032.631+80131.659X	0.993	0.0625-4

#### Lung targeting of SPA-DXM-NLP

**Figure 3:**
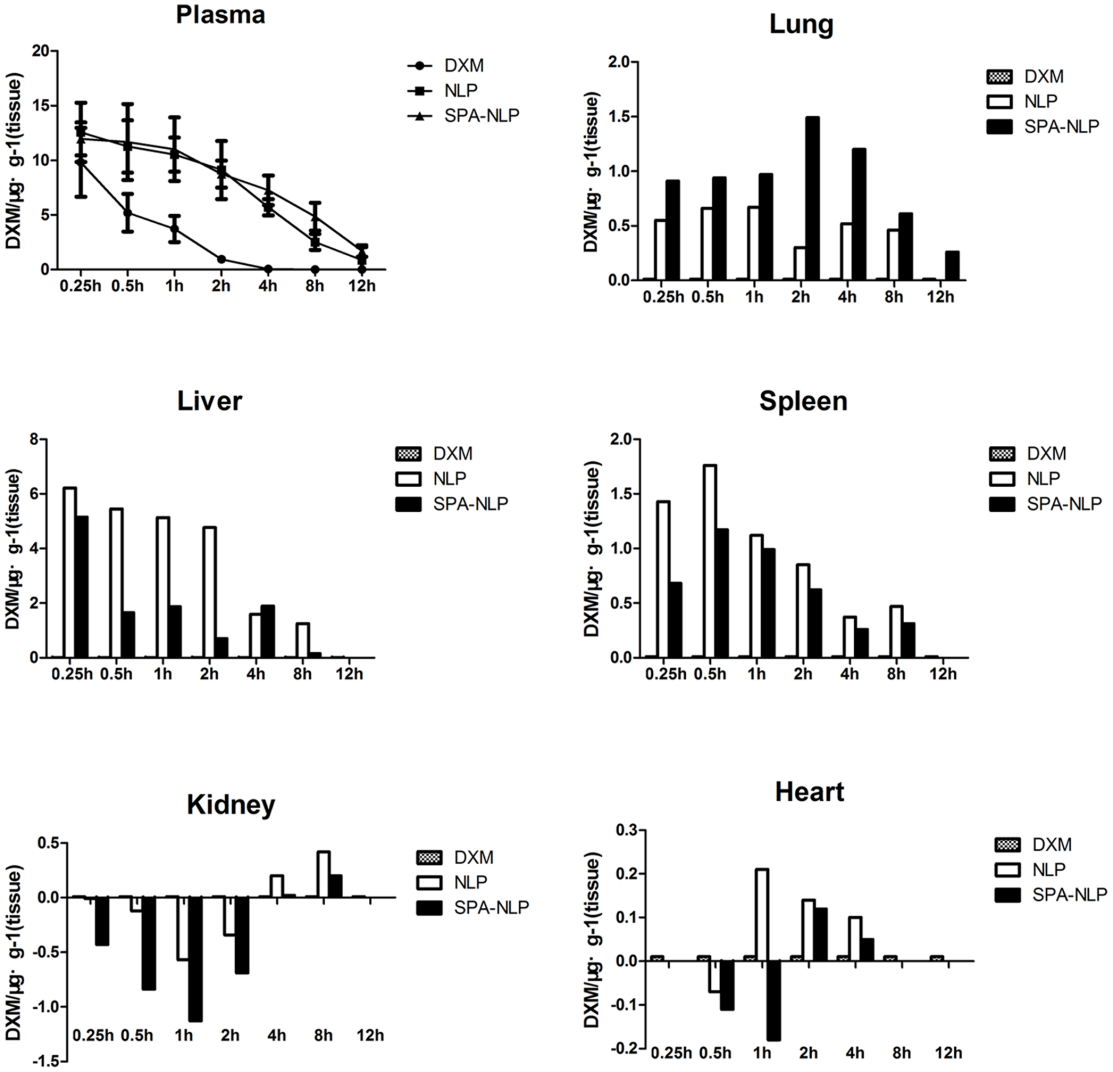
Dexamethasone sodium phosphate (DXM) distribution in plasma and tissues after intravenous administration of DXM, DXM-NLP and SPA-DXM-NLP. a) The residence time of DXM-NLP and SPA-DXM-NLP in the circulation was prolonged and blood drug concentrations of SPA-DXM-NLP were significantly higher than that of DXM. b) DXM’s distribution in different tissues after intravenous administration of DXM, DXM-NLP and SPA-DXM-NLP. *Y* value stands for the relative concentration of DXM.SPA-DXM-NLP showed clear evidence of lung targeting.

Figure 3 shows the concentrations of dexamethasone sodium phosphate in the plasma of rats and the mean DXM tissue concentrations at different sampling time points following intravenous injection with SPA-DXM-NLP, DXM-NLP and DXM. The persistence of DXM-NLP and SPA-DXM-NLP in blood was longer than that of DXM. Blood concentrations of SPA-DXM-NLP were significantly higher than those of DXM at 0.5, 1, 2, 4, 8 and 12 h post injection, (*P*<0.05).

Dexamethasone release from immunoliposomes will occur due to either osmotic differences between the liposome lumen and the surrounding environment, or due to breakdown of the liposome structure which will lead to rapid release. This may eventuate in the blood as well as tissues. Once taken up by cells, the immunoliposomes will also release their cargo as their structural integrity is degraded.

Concentrations of DXM in groups treated with DXM-NLP and SPA-DXM-NLP were significantly higher than those in the DXM-treated group at (*P*<0.05). The difference in lung tissue was most pronounced in the SPA-DXM-NLP group, whereas the increases in liver and spleen were higher in the DXM-NLP than in the SPA-DXM-NLP groups. At time points up to 2 h, post administration, concentrations of dexamethasone sodium phosphate in the kidney were significantly lower with SPA-DXM-NLP than with DXM injection (*P*<0.01). However, higher concentration was observed in the SPA-DXM-NLP group at 8 h (*P*<0.01). No significant differences in dexamethasone sodium phosphate concentrations were observed in the heart with any of the three preparations (*P*>0.05).

**Figure 4:**
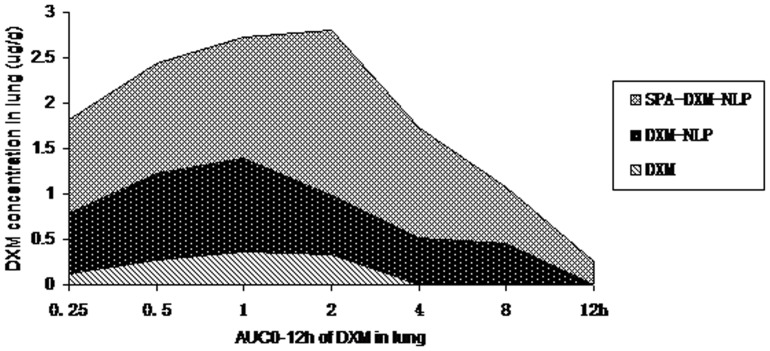
AUC_0–12__h_ of DXM in lung following administration of DXM, DXM-NLP and SPA-DXM-NLP. AUC_0–12__h_ was 11.26 µg/g in the SPA-DXM-NLP group and 0.28 µg/g in the DXM group (*R*_e_  = 40.21).

The peak concentration ratio (*C*_*e*_) and relative rate of uptake (*R*_*e*_) were used to investigate the targeting of SPA-DXM-NLP and DXM-NLP in different rat tissues (Figure 4 and Table 2). The results revealed significant targeting of SPA-DXM-NLP to lung tissue. The peak lung concentration of dexamethasone sodium phosphate following injection of SPA-DXM-NLP (1.83 µg/g) was 5.1-times higher than that achieved with DXM injection (0.36 µg/g; *C*_*e*_  = 5.1). The area under concentration-time curve 12 h after injection with SPA-DXM-NLP (11.26 µg/g) was 40.2-times than that in the DXM group (0.28 µg/g; *R*_*e*_  = 40.2).

**Table 2:** Target evaluation of DXM-NLP and SPA-DXM-NLP.

Sample	Relative Rate of Uptake		Peak Concentration Ratio	
	DXM-NLP	SPA-DXM-NLP	DXM-NLP	SPA-DXM-NLP
Plasma	2.9	3.9	1.3	1.2
Lung	24.7	40.2	2.9	5.1
Liver	2.0	1.4	1.8	1.5
Spleen	4.4	3.4	4.9	3.6
Kidney	1.3	0.7	0.8	0.4
Heart	1.3	1.0	1.4	0.9

*R*_*e*_: the relative rate of uptake;

*C*_*e*_: peak concentration ratio.

Values for *R*_*e*_ and *C*_*e*_ for SPA-DXM-NLP in heart and kidney tissue were less than 1, indicating no targeting to these tissues.

### Therapeutic Effect of SPA-DXM-NLP on Bleomycin-induced Acute Lung Injury in Rats

#### Pathological changes of lung tissues of rats

**Figure 5:**
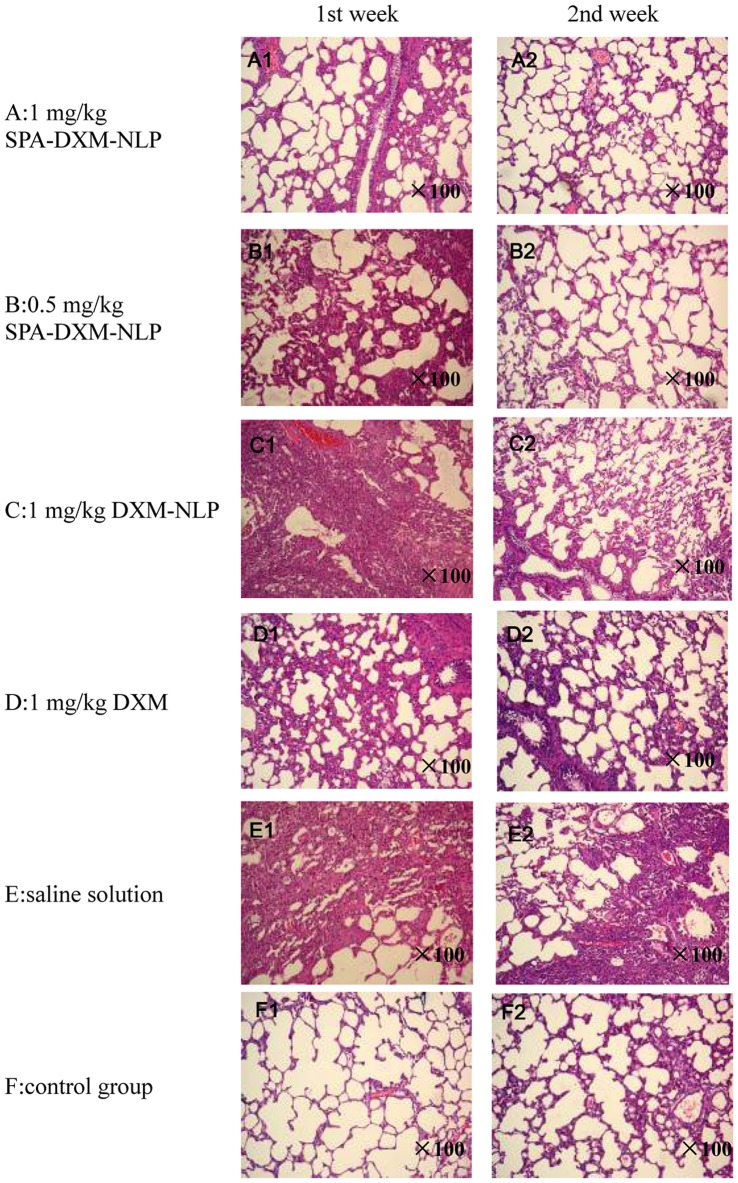
H&E staining of lung samples following treatment with SPA-DXM-NLP, DXM-NLP, DXM and saline solution (×100).

H&E staining of the pathological samples of lung tissues showed evidence of an inflammatory response 1 week after treatment with bleomycin, with alveolar wall thickening and alveolar interval widening. Infiltration of a large number of inflammatory cells with mass pulmonary consolidation was observed in untreated ALI rats (Figure 5 E1). The inflammatory response was ameliorated following administration of dexamethasone sodium phosphate (Figure 5 D1). Lung injury was significantly improved in rats treated with SPA-DXM-NLP (Figure 5 A1 and B1). However, there were no statistically significant differences in semi-quantitative scores(Table 3) of lung injury between rats treated with SPA-DXM-NLP, DXM-NLP, DXM and untreated ALI (*P*>0.05).

**Table 3:** Acute lung injury scores.

Group	1 week later	2 weeks later
	ALI scores	ALI scores
A	11.4 (7–14)	7.6 (4–11)*
B	12.2 (8–14)	11 (7–13)
C	14.2 (13–16)	12.6 (8–15)
D	13.8 (12–16)	13.2 (11–15)
E	15.4 (14–16)	15.75 (15–16)
F	0	0.4 (0–1)

* *P*<0.05 vs. Group E. Group A: SPA-DXM-NLP (1 mg/kg); Group B: SPA-DXM-NLP (0.5 mg/kg); Group C DXM-NLP; Group D: DXM; Group E: untreated ALI rats; Group F: normal controls.

At 2 weeks, the lung inflammatory response remained severe in the untreated ALI rats (Figure 5 E2). There was no significant improvement in lung injury in rats treated with DXM relative to Week 1 (Figure 5 D2). The lung injury in rats treated with DXM-NLP became lighter (Figure 5 C2), whereas continued improvement relative to Week 1 was observed in rats receiving SPA-DXM-NLP (Figure 5 A2 and B2). At 2 weeks lung injury scores in rats treated with SPA-DXM-NLP (1 mg/kg) were significantly higher than in untreated ALI rats (P  = 0.026). Figure 5 F1 and F2 represent the control group at 1 and 2 weeks and compare favorably with Figure 5 A2.

#### TNF-αand TGF-β1 levels in BALF

**Figure 6:**
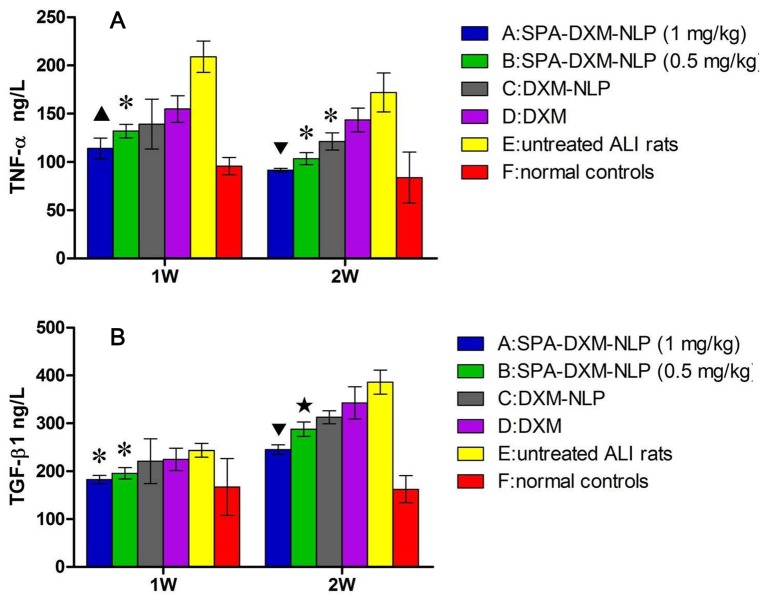
TNF-α and TGF-β1 levels in BALF, determined by enzyme-linked immunosorbent assay (ELISA, R&D Systems,US). A) TNF-α levels in BALF**:** ▴ *P*<0.05 vs. Groups B, D and E; * *P*<0.05 vs. Groups D and E; ▾ *P*<0.05 vs. Groups B, C, D and E. **B)** TGF-β1 levels in BALF **:** * *P*<0.05 vs. Groups D and E; ▾ *P*<0.05 vs. Groups B, C, D and E; ★ *P*<0.05 vs. Groups A, C, D and E.

Cytokines levels in BALF, indicators of inflammation, are shown in Figure 6. One week after bleomycin administration, levels of TNF-αand TGF-β1 in BALF were significantly higher in untreated ALI rats than in controls (*P*<0.05). At Week 2, the level of TNF-α was lower than that at Week 1, whereas TGF-β1 levels were significantly increased (*P*<0.05).

At Weeks 1 and 2 post-treatment, TNF-α levels were significantly lower in rats treated with SPA-DXM-NLP than in untreated rats or in those treated with DXM-NLP (*P*<0.05). Significant differences were also observed between rats treated with different doses of SPA-DXM-NLP (Figure 6A; *P*<0.05).

Two weeks after bleomycin injection, levels of TGF-β1 in BALF significantly increased in all rats except the control group. At 1 and 2 weeks post treatmentTGF-β1 levels were significantly lower in rats receiving SPA-DXM-NLP than untreated rats or rats receiving DXM (*P*<0.05). There were also significant differences in the levels of this cytokine at Week 2 between rats receiving 0.5 or 1 mg/kg SPA-DXM-NLP and DXM-NLP (*P*<0.05; Figure 6B).

#### Bacterial and fungal culture in BALF

One week post-treatment with bleomycin, there were five cases of infection. These included one case of Gram-positive bacilli in the DXM-NLP group and three cases in the DXM group. There was also a single case of *Staphylococcus epidermidis* in the untreated ALI group.

#### Survival

**Figure 7:**
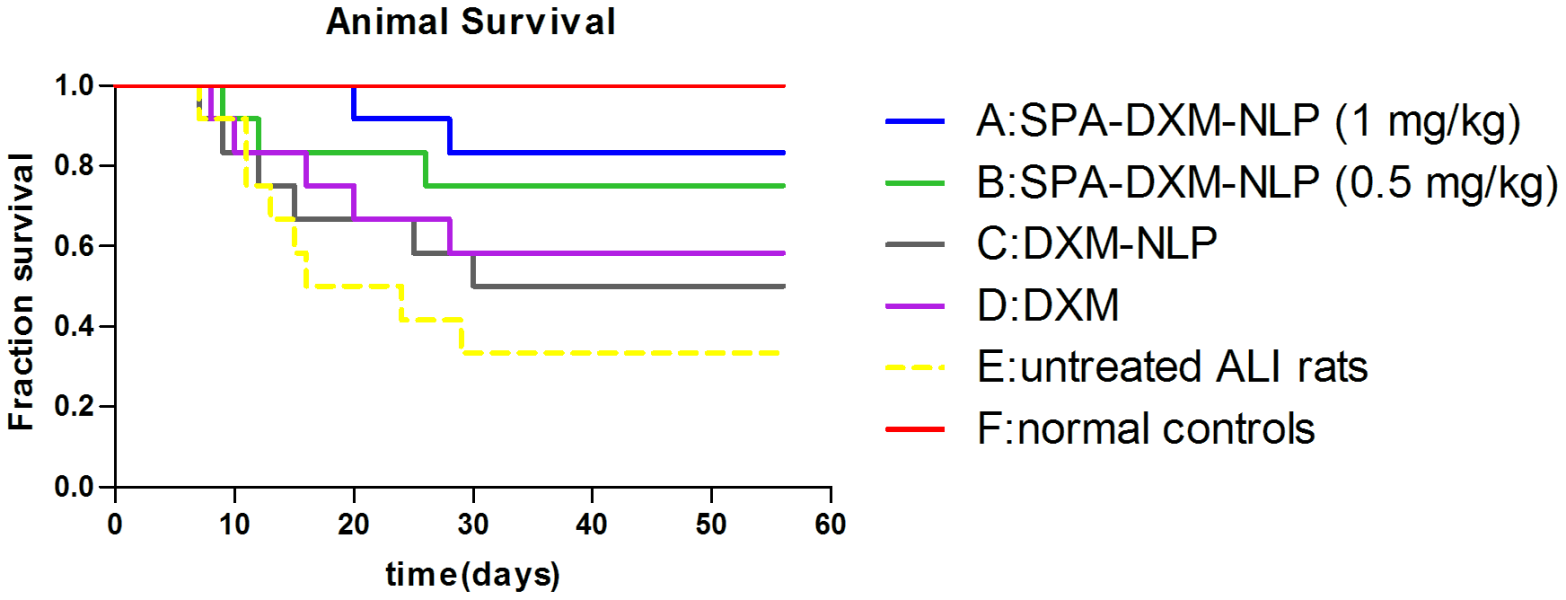
Survival curves. Survival rate of rats treated with SPA-DXM-NLP (1 mg/kg) was 83.3% compared with a survival rate of 33.3% in untreated ALI rats (*P*<0.05).

Survival curves 8 weeks post-treatment with 10 mg/kg bleomycin are shown in Figure 7. Survival in rats treated with 1 mg/kg SPA-DXM-NLP (83.3%) was significantly higher than that in untreated ALI rats (33.3%; *P*<0.05). No statistically significant differences in survival were observed between untreated rats and those receiving SPA-DXM-NLP (0.5 mg/kg), DXM-NLP or DXM (*P*>0.05), nor between any of these three active treatment groups (Table 4). The survival of rats receiving SPA-DXM-NLP (1 mg/kg) was numerically higher than in rats treated with DXM, but this difference was not statistically significant.

**Table 4:** Survival of rats in different groupsafter 8-week observation.

Group	Number of rats	Survival rate (%)
A	12	83.3*
B	12	75
C	12	50
D	12	58.3
E	12	33.3
F	12	100

* *P*<0.05 vs. Group E. Group A: SPA-DXM-NLP (1 mg/kg); Group B: SPA-DXM-NLP (0.5 mg/kg); Group C DXM-NLP; Group D: DXM; Group E: untreated ALI rats; Group F: normal controls.

## Discussion

In the present study, we prepared thin-lipid films which were dispersed in mild conditions (no ultrasonication used) in combination with extrusion methods to prepare monodisperse liposomes, in which to encapsulate dexamethasone sodium phosphate for targeted drug delivery. Drug encapsulation efficiency was more than 90% and good stability was observed after 4 weeks of storage.

In general terms, liposome targeting can be achieved through processes of active or passive targeting. The pharmacokinetics and tissue distribution of liposomes following intravenous injection are determined by many variables, such as the size, composition, stealth corona, targeting component and health status of the body [24], [25]. If phagocytosis cannot be reduced through the presence of a stealth corona or poly(ethylene glycol) (PEG) layer, intravenously administered liposomes will be removed from circulation rapidly and gather in liver Kupffer cells and splenic macrophages, thereby exhibiting passive targeting to liver and spleen [26]. For lung specific drug delivery vehicles such as the liposomes presented here it is therefore important to reduce liver and spleen uptake in order to achieve maximal targeted delivery to the lung. Small liposomes (<100 nm) have been shown [13] to reduce phagocytic uptake within the liver and spleen, however, liposomes that are too small cannot be adequately captured by lungs. We therefore used liposomes with diameters of approximately 130 nm in the current study.

The highly hydrophilic nature of the PEG layer that surrounds the lioposome, also known as the stealth corona, forms a steric barrier at the liposome surface, which prevents plasma protein and opsonin binding. This reduces phagocytosis, and thereby prolongs circulation time. Surface modification of liposome with PEG also increases steric hindrance between lipsomes, preventing their flocculation [27]. In our study using PEG as a liposome protection agent to significantly prolonged the blood residence time of DXM-NLP and SPA-DXM-NLP to approximately 12 h compared to a residence time of only 4 h for dexamethasone sodium phosphate.

Crosslinking agents are used to join antibodies to liposomes that contain biologically active groups and form immunoliposomes. The agents used to crosslink liposomes and antibodies include glutaraldehyde, carbodiimide and SPDP [21], [28]. SPDP is an ideal crosslinking agent which is activated using mild reaction conditions. It is associated with few adverse effects, minimal crosslinking between antibodies and has been successfully used in the preparation of many immunoliposomes [10], [11], [21]. Yokoe and colleagues [6] coupled recombinant human serum albumin (rHSA) with doxorubicin (DXR) liposome to prepare rHSA/PEG liposomal DXR using SPDP as a crosslinker. This was shown to increase the efficacy and safety of PEG liposomal DXR in tumor-bearing rats. Betageri *et al* [11]. conjugated a mouse monoclonal antibody IgG2a with the liposome carrying an anti-human immunodeficiency virus (HIV) drug, dideoxycytidine triphosphate (ddCTP) using SPDP as a heterobifunctional reagent. This process significantly increased drug uptake into human monocyte/macrophages.

In the present study, DSPE-PEG2000-PDP which contains a PDP group was added during liposome preparation enabling the procedure to be further simplified compared with the classical liposome preparation method using SPDP.

Active targeting of DXM-NLP in lung was achieved through the specific binding of SP-A antibody to SP-A in lung tissues. The results showed that SPA-DXM-NLP significantly increased the concentration of dexamethasone sodium phosphate and prolonged its residence time in lung tissue. The peak concentration of dexamethasone sodium phosphate post-injection with SPA-DXM-NLP was 5-times higher than that achieved with dexamethasone sodium phosphate and the 0–12 h area under the concentration-time curve was 40.–times higher indicating specific lung targeting thought the use of active targeting.

This specific targeting provides an opportunity for decreasing glucocorticoid dosage, enhancing therapeutic efficacy in and reducing systemic adverse reactions in clinical practice. Further studies will be required to investigate the distribution density of the liposome surface antibody, to determine the coupling efficiency between liposome and antibody, and investigate ways of increasing drug targeting efficiency of immunoliposomes. The effect of prolonged administration on immune response will also need to be assessed. The anti-inflammatory and immunosuppressive effects of glucocorticoid have been widely used for the treatment of ALI/ARDS to prevent excessive inflammatory reactions and protect the functional integrity of the lung [29]–[36]. The therapeutic use of these agents remains controversial in terms of dosage, duration of treatment and the improvement in mortality [3], [4], [18], [29]–[38]. Severe adverse effects are mainly caused by the nonselective distribution of the drug in the body, which also results in inadequate concentrations of drug in diseased organs and tissues. The actively-targeted liposomes presented in this study arean effective method of delivering glucocorticoids to lung inflammation sites thus reducing drug distribution to healthy tissues, and significantly increasing the benefit-risk ratio.

In the present study, we used lung-targeted SPA-DXM-NLP to treat bleomycin-induced acute lung injury in rats. The results indicated that SPA-DXM-NLP (1 mg/kg) significantly alleviated the pathological damage to lung tissues and reduced mortality compared with normal dexamethasone sodium phosphate preparation. SPA-DXM-NLP also reduced the levels of TNF-α and TGF-β1 in BALF of rats with lung injury. TGF-β1 is a key cytokine in the process of fibrogenesis [39]. It is well known that TGF-β1 promotes the proliferation and differentiation of fibroblasts into activated myofibroblasts, and also promote epithelial mesenchymal transition which is another source of myofibroblasts., TGF-β1 also enhances collagen and fibronectin production and reduces extracellular matrix degradation [40].

Currently, the only way to increase the concentration of the dexamethasone sodium phosphate in lung tissue is to increase the systemic use dosage, which in turn markedly increases the risk of adverse effects such as secondary infections, osteoporosis, diabetes and hypertension. However, our results show that the therapeutic efficacy of a lower dosage of 0.5 mg/kg SPA-DXM-NLP was superior to that of normal dexamethasone sodium phosphate (1.0 mg/kg), suggesting that targeted SPA-DXM-NLP was able to increase the local concentration of dexamethasone and prolong its residence time in lung tissue. But there was still a large amount of DXM was detected in the liver and spleens of treated rats, That’s mainly because immunoliposome was a particle size in ±140 nm, its was easily be cleared by the phagocytes in liver and spleen, which was inevitable. So the concentration of DXM in livers and spleens were not lower.

The survival of rats receiving SPA-DXM-NLP (1 mg/kg) was numerically higher than in rats treated with DXM.Why there were no statistically significant in survival between untreated rats and those receiving SPA-DXM-NLP (0.5 mg/kg), DXM-NLP or DXM, we considered that might be related to the less number of model animals. Hence, we will increase the number of animals in following study.

One of the main purposes of targeted hormone therapy is to reduce the adverse effects associated with high dose, long-term use. In the present study, no bacteria were isolated from rats treated with SPA-DXM-NLP. These findings indicate that SPA-DXM-NLP might decrease the occurrence of infectious complications possibly by improving overall immune status as a result of amelioration of lung injury.

### Conclusion

The preparation of actively targeted dexamethasone loaded lipsomes was successfully carried out and their in vivo performance was assessed. The current study showed clear evidence that using SPA-DMX-NLP to deliver dexamethasone to the lung was associated with a better therapeutic effect and fewer adverse effects than the normal dexamethasone sodium phosphate in rats with bleomycin-induced acute lung injury These findings provide a scientific and experimental basis for targeted therapy of lung diseases with glucocorticoid drugs.
